# Upregulation of keratin 15 is required for varicella-zoster virus replication in keratinocytes and is attenuated in the live attenuated vOka vaccine strain

**DOI:** 10.1186/s12985-024-02514-8

**Published:** 2024-10-09

**Authors:** Cristina Tommasi, Ohad Yogev, Michael B. Yee, Andriani Drousioti, Meleri Jones, Alice Ring, Manuraj Singh, Inga Dry, Oscar Atkins, Aishath S. Naeem, Nisha Kriplani, Arne N. Akbar, Jürgen G. Haas, Edel A. O’Toole, Paul R. Kinchington, Judith Breuer

**Affiliations:** 1 Infection, Immunity and Inflammation Department, University College London GOS Institute of Child Health, London, UK; 2 Infection and Immunity Department, University College London, London, UK; 3 Department of Ophthalmology and of Molecular Microbiology and Genetics, University of Pittsburgh School of Medicine, Pittsburgh, US; 4 Dermatology, St. George’s Hospital, London, UK; 5 Infection Medicine, University of Edinburgh, Edinburgh, UK; 6 Experimental & Translational Medicine, Division of Medicine, University College London, London, UK; 7 Centre for Cell Biology and Cutaneous Research, Blizard Institute, Queen Mary University of London, London, UK; 8 Eleven Therapeutics, Cambridge, UK; 9 Krystalbio Inc, Pittsburgh, US; 10 UKHSA, Porton Down, UK; 11 The Roslin Institute, Edinburgh, UK; 12 Francis Crick Institute, London, UK; 13 Dana-Farber Cancer Institute, Boston, US

**Keywords:** VZV, KRT15, IE62, vOka vaccine

## Abstract

Varicella-zoster virus (VZV) is the etiological agent of chickenpox and shingles, diseases characterised by epidermal virus replication in skin and mucosa and the formation of blisters. We have previously shown that VZV infection has a profound effect on keratinocyte differentiation, altering the normal pattern of epidermal gene expression. In particular, VZV infection reduces expression of suprabasal keratins 1 and 10 and desmosomal proteins, disrupting epidermal structure to promote expression of a blistering phenotype. Here, we extend these findings to show that VZV infection upregulates the expression of keratin 15 (KRT15), a marker expressed by basal epidermal keratinocytes and hair follicles stem cells. We demonstrate that KRT15 is essential for VZV replication in the skin, since downregulation of KRT15 inhibits VZV replication in keratinocytes, while KRT15 exogenous overexpression supports viral replication. Importantly, our data show that VZV upregulation of KRT15 depends on the expression of the VZV immediate early gene ORF62. ORF62 is the only regulatory gene that is mutated in the live attenuated VZV vaccine and contains four of the five fixed mutations present in the VZV Oka vaccine. Our data indicate that the mutated vaccine ORF62 is not capable of upregulating KRT15, suggesting that this may contribute to the vaccine attenuation in skin. Taken together our data present a novel association between VZV and KRT15, which may open a new therapeutic window for a topical targeting of VZV replication in the skin via modulation of KRT15.

**Supplementary Information:**

The online version contains supplementary material available at 10.1186/s12985-024-02514-8.

## Introduction

Replication in skin is essential to the pathogenesis of varicella-zoster virus (VZV), a human alphaherpesvirus that causes varicella (chickenpox) upon primary infection and zoster (shingles) following reactivation from a neuronal latent state [1]. The formation of fluid-filled skin vesicles, rich in cell-free infectious virus, is important for transmission of virus to naïve hosts through aerosol-mediated spread and inhalation [2]. Cell-free virus is also required for infection of sensory nerve endings in the skin, where retrograde transport of virus to sensory neuronal bodies permits VZV to establish persistence and latency [2]. The live attenuated vOka vaccine is widely used to prevent varicella and, at higher doses, shingles, and is attenuated for replication in human skin, while seemingly remaining replication competent in other target tissues such as peripheral blood lymphocytes and neuronal tissue [3, 4].

The outermost layer of the skin, the epidermis, is largely comprised of specialised epithelial cells known as keratinocytes, which undergo a program of terminal differentiation as they migrate towards the epidermal surface, transitioning from basal undifferentiated cells to differentiated anuclear corneocytes that are sloughed off [5]. Each epidermal stratum is associated with a distinct pattern of gene expression; as keratinocytes differentiate, they lose basally expressed proteins including keratins 5 and 14 and upregulate expression of terminal differentiation markers [6, 7]. During primary infection, VZV infects the stem cells in the hair follicle, spreading to the skin basal keratinocytes, although the precise mechanisms involved are still unknown [8]. Thereafter, VZV replication in skin becomes stratum-dependent, with full lytic replication occurring as infected keratinocytes differentiate [9]. Our previous RNAseq analyses indicate that VZV replication in keratinocytes is associated with dysregulation of normal cellular gene expression [9]. The virus drives a pattern of expression similar to that seen in blistering disorders, in which there is downregulation of the suprabasally expressed keratin 10 [9, 10]. This is also accompanied by loss of epidermal cell-cell junctions, promoting the formation of syncytial structures [9–11]. At the same time, we noted that VZV infection of keratinocytes upregulates the expression of keratin 15 (KRT15), which is normally expressed by stem cells in the hair follicles, as well as by basal undifferentiated keratinocytes in the interfollicular epidermis [7, 9]. Notably, other markers of basal undifferentiated keratinocytes, including keratin 5 and keratin 14, remained unchanged by VZV infection, suggesting that VZV-induced upregulation of KRT15 is specific [9].

Here, we show that KRT15 is required for VZV replication and this is mediated by the major VZV transcriptional transactivating protein IE62. Our finding that vaccine strain vOka IE62, which harbours four of the five vaccine mutations that are fixed, fails to upregulate KRT15, provides the first indication that this may be a contributory mechanism to vaccine attenuation.

## Results

### VZV infection leads to KRT15 upregulation

**Fig. 1:**
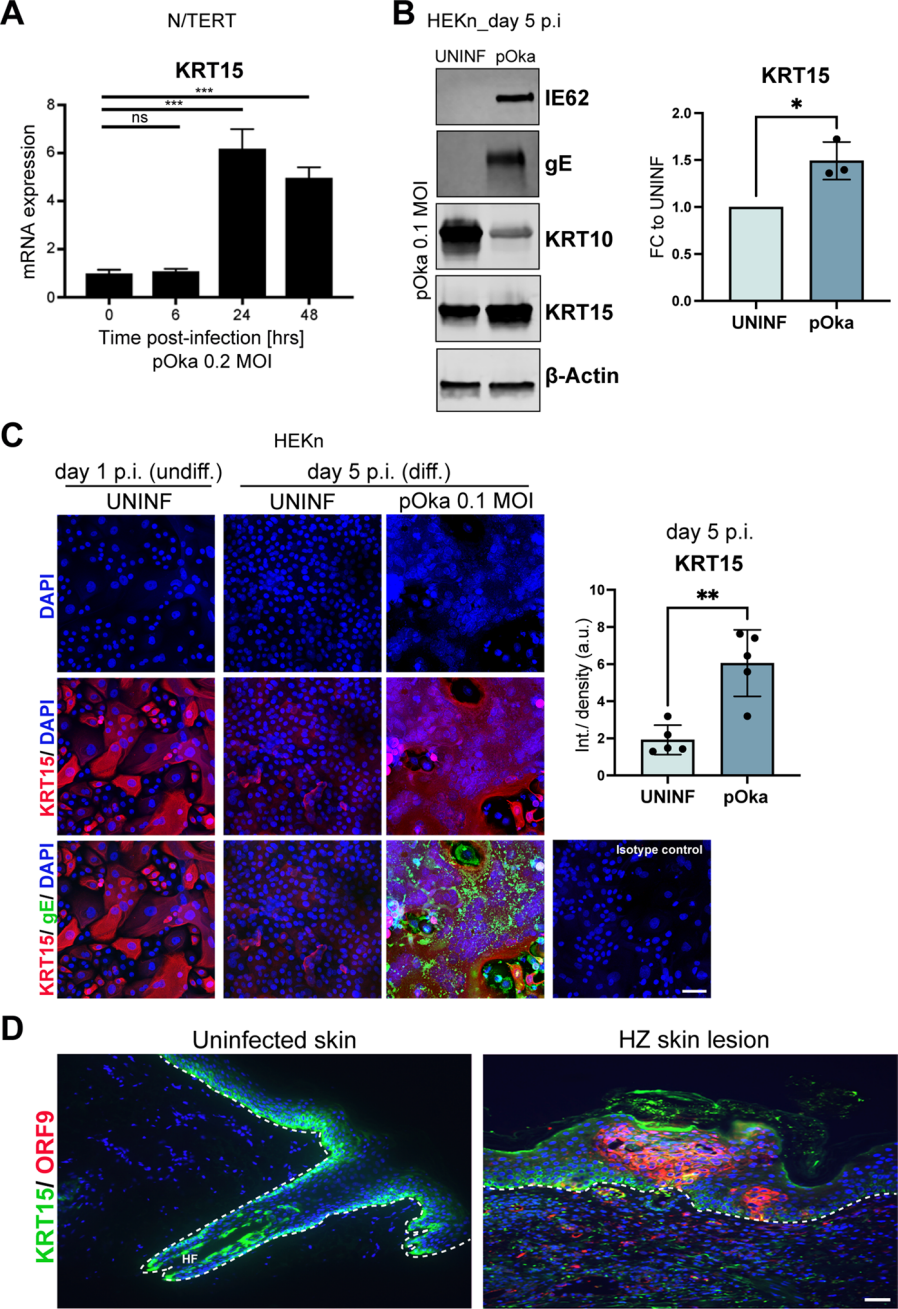
VZV infection increases KRT15 expression. **(A)** N/TERT keratinocytes were infected with cell-free VZV parental strain pOka at an MOI of 0.2 and induced to differentiate at day 1 post-infection. Infected N/TERTs were then tested at the indicated time points for KRT15 mRNA expression by quantitative reverse transcription PCR (qRT-PCR). Tubulin beta (*TUBB*) levels were used for normalization for RNA signal input. *n* = 3 independent experiments. Error bars: SD. **(B)** HEKn keratinocytes were infected with pOka at an MOI of 0.1 for 5 days. HEKn were induced to differentiate at day 3 post-infection and left in culture for 2 further days before being collected. Protein levels were analysed by Western blotting. KRT15 densitometry was quantified and normalised to β-actin in *n* = 3 independent experiments and presented as fold change (FC) to UNINF. Error bars: SD. **(C)** KRT15 expression as visualised by immunofluorescence in HEKn keratinocytes infected with pOka at an MOI of 0.1 for 5 days. HEKn were induced to differentiate at day 3 post-infection and left in culture for 2 further days before being fixed. Infection with pOka is indicated by the expression of the VZV gE protein, as well as by the presence of syncytia structures displayed in the DAPI channel. Images shown are representative of *n* = 2 independent experiments. Graph shows average quantification of integrated density of KRT15 signal relative to number of nuclei in 5 fields of view for each of the UNINF and pOka conditions at day 5 p.i. Error bars: SD. **(D)** KRT15 and ORF9 protein expression as visualised by immunofluorescence in uninfected skin (displaying hair follicle, HF) and in vesicular skin lesion in herpes zoster (HZ). Images are representative of *n* = 4 patients skin samples analysed per condition. Dotted lines mark the epidermal-dermal junction. Scale bar, 50 μm. Statistical significance was calculated by one-way ANOVA and two-tailed t test **(** and denoted by *P < 0.05, **P < 0.01,***P < 0.001. ns, not significant; hrs, hours; UNINF, uninfected; p.i., post infection; undiff, undifferentiated; diff, differentiated; SD, standard deviation

To confirm the upregulation of KRT15 by VZV infection, we first infected immortalised N/TERT keratinocytes with cell-free wild-type VZV (pOka strain) at MOI 0.2 and tested the expression levels of KRT15 over time. We observed significant increase in KRT15 mRNA levels at 24 and 48 h hours post-infection (Fig. 1A). Similarly, immunofluorescence staining of immortalised N/TERT keratinocytes shows increased levels of KRT15 three days post-VZV infection (Supplementary Fig. S1). To confirm this in primary keratinocytes, we infected neonatal human epidermal keratinocytes (HEKn) with pOka at MOI 0.1. Increase in KRT15 was observed by Western Blotting in HEKn infected with pOka, as corroborated by expression of the VZV IE62 and gE proteins in the infected cells, as well as substantial KRT10 decrease as previously reported [9, 10] (Fig. 1B). We further investigated this by immunofluorescence staining in order to examine the expression of KRT15 specifically in the infected cells. As expected from the previously reported KRT15 expression patterns [9], KRT15 is decreased in the uninfected and differentiated HEKn compared to the undifferentiated, while in the cells positive for the VZV gE protein, and characterised by the typical VZV syncytia structures, KRT15 is highly increased (Fig. 1C).

Finally, the upregulation of KRT15 was confirmed in skin biopsies from herpes zoster patients; normally KRT15 expression is largely confined to the basal layer of the epidermis, whereas in the VZV-infected skin, and particularly in the lesion region, KRT15 expression is widespread in all the epidermal layers. (Fig. 1D).

**Fig. 2:**
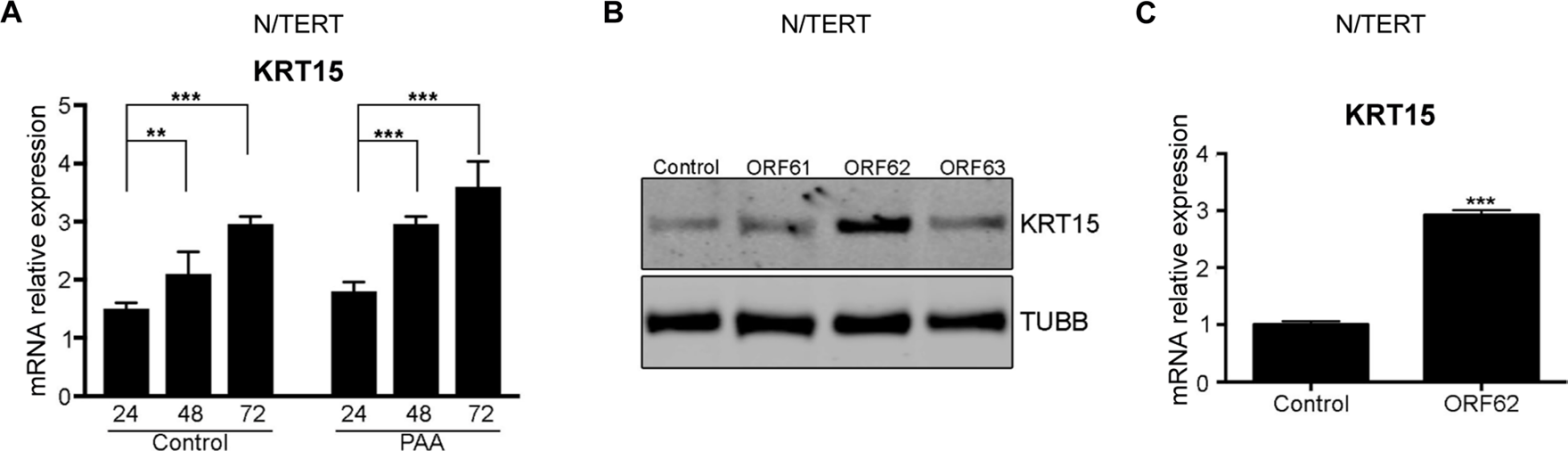
VZV immediate early gene ORF62 induces KRT15 upregulation. **(A)** PAA-treated or control-treated N/TERT keratinocytes were harvested at the indicated time points (hrs post-infection) and analysed for KRT15 mRNA levels by qRT-PCR. KRT15 mRNA levels were normalised to *TUBB* levels. Error bars: SD (*n* = 3 experiments). **(B)** KRT15 protein levels detected by Western blotting were analysed in N/TERT keratinocytes transduced to overexpress the VZV transcriptional regulatory proteins; ORF61, ORF62 or ORF63. **(C)** KRT15 mRNA levels were analysed by qRT-PCR in N/TERT keratinocytes overexpressing ORF62 as compared to control. KRT15 mRNA levels were normalised to *TUBB* levels. Error bars: SD (*n* = 3 experiments). Statistical significance was calculated by one-way ANOVA in **(A)** and two-tailed t test in **(C)** and denoted by **P < 0.01, ***P < 0.001. SD, standard deviation

To further understand how VZV upregulates KRT15 expression, we first tested whether viral replication is required. To do this, we added phosphonoacetic acid (PAA) at the time of a cell-free initiated infection, which inhibits the viral DNA polymerase and therefore transcription of the viral late genes, while not affecting the expression of the immediate early (IE) genes ([12] and Supplementary Fig. S2). Treatment of N/TERT keratinocytes with PAA did not alter KRT15 upregulation suggesting that the expression of immediate early genes may be more important than full viral replication for this phenotype (Fig. 2A). To test this and to identify which of the IE genes may be responsible for this upregulation, we transduced N/TERT keratinocytes with lentiviral vectors expressing each of three IE genes, ORF61, 62 and 63. Only expression of ORF62 resulted in upregulation of KRT15 (Fig. 2B and C). Taken together, these results show that VZV infection upregulates KRT15 expression and that this appears to depend on the expression of the IE gene ORF62.

### KRT15 is required for VZV epidermal replication

**Fig. 3:**
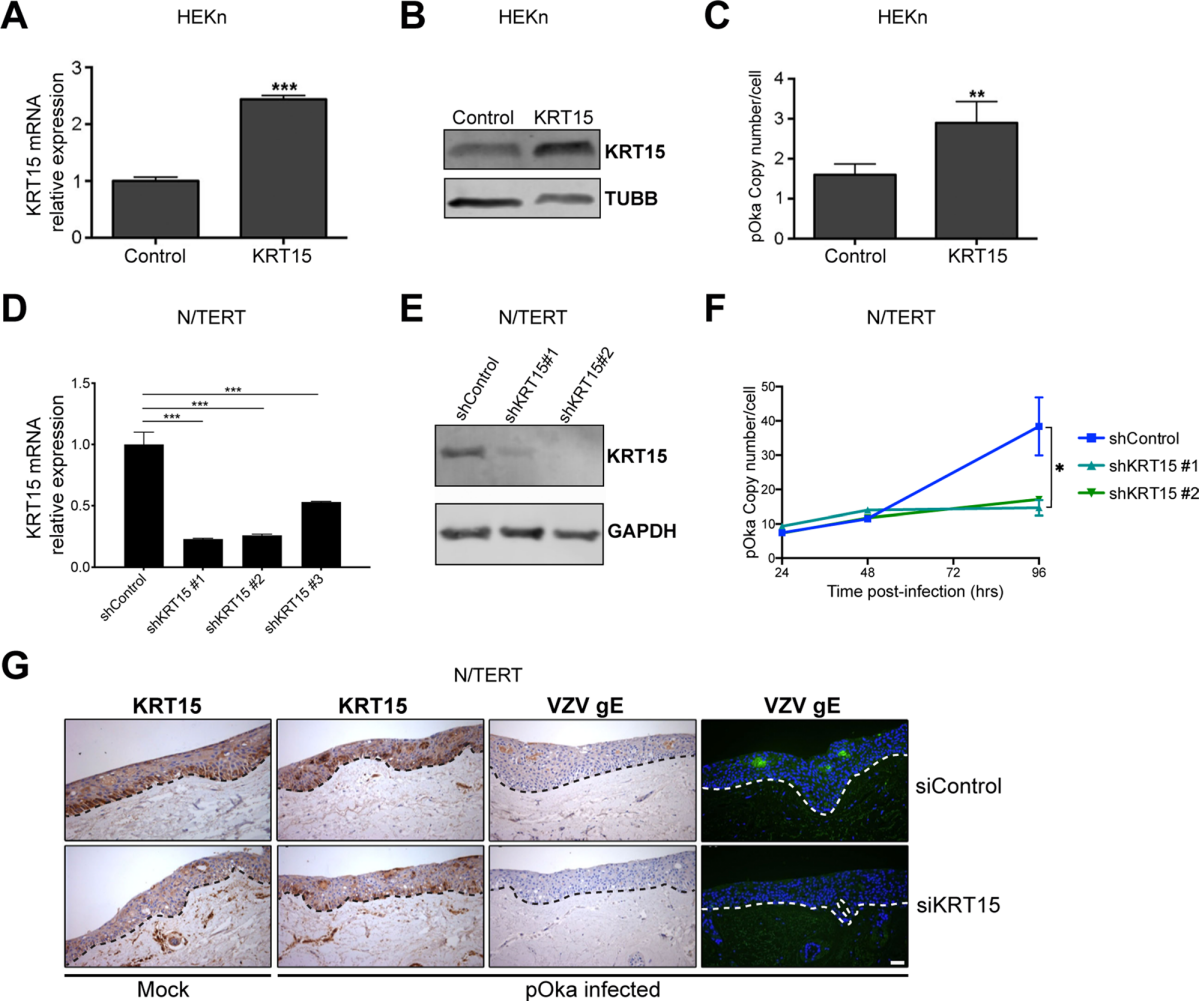
KRT15 upregulation supports VZV replication. **(A**,** B)** Transduced HEKn overexpressing the KRT15 gene were analysed for KRT15 **(A)** mRNA by qRT-PCR and **(B)** protein levels by Western Blotting. Graph in **(A)** is average quantification of *n* = 3 biological replicates ± SD. **(C)** KRT15-overexpressing HEKn were then infected with VZV pOka and analysed by qPCR for VZV genome copy number and normalized per cell. Error bars: SD (*n* = 3 independent experiments). **(D**,** E)** N/TERT keratinocytes were infected with lentivirus expressing KRT15 specific short hairpin RNAs (shRNAs). 72 h post-infection cells were analysed for KRT15 **(D)** mRNA by qRT-PCR and **(E)** protein levels by Western Blotting. Graph in **(D)** is average quantification of *n* = 3 biological replicates ± SD. **(F)** Cells were harvested at the indicated time points post-VZV (pOka) infection and analysed for VZV genome copy number per cells using qPCR. Error bars: SD (*n* = 3 independent experiments). **(G)** Organotypic rafts of control and KRT15 knockdown (by means of siRNA) N/TERT keratinocytes were mock-infected or infected with VZV pOka nine days post-lifting at the air-liquid interface. Five days post-infection, the rafts were fixed and stained using antibodies for KRT15 or the viral protein VZV gE. Dotted lines mark the epidermal-dermal junction. Data is representative of biological triplicates and representative of results obtained in *n* = 2 independent experiments. Statistical significance was calculated by two-tailed t test **(A)** and one-way ANOVA **(D** and **F)** and denoted by *P < 0.05, **P < 0.01, ***P < 0.001. Scale bar, 50 μm. SD, standard deviation

We next investigated the influence of KRT15 levels on VZV epidermal replication. We first overexpressed KRT15 in HEKn using lentiviral vectors (Fig. 3A and B), following which, we infected the transduced cells with cell-free VZV and assessed viral replication through quantification of viral DNA copy number. When KRT15 was overexpressed 48 h prior to VZV infection, the virus replication levels were significantly increased over the levels in cells transduced with an empty lentiviral vector (Fig. 3C). This suggests that KRT15 expression is proviral.

We then tested the effect of KRT15 knockdown on VZV replication. For this, N/TERT keratinocytes were infected with lentiviruses expressing each of three specific shRNAs designed to target different sequences on the KRT15 mRNA. Knockdown of KRT15 mRNA by shKRT15 #1 and #2 reduced KRT15 mRNA levels the most efficiently, reducing it to about 25% of the control, while shKRT15 #3 had less of a silencing effect, reducing KRT15 to approximately ∼50% mRNA levels compared to cells expressing a scrambled shRNA control (Fig. 3D). The shKRT15 #1 and #2 also had a corresponding significant effect on the KRT15 protein levels in the cells (Fig. 3E). We next examined the levels of KRT15 in these cells in response to VZV infection. For this, KRT15 mRNA levels were monitored in the knockdown and control cells post-VZV infection at 48 and 96 h by qRT-PCR. While shKRT15 #1 and #2 prevented the upregulation of KRT15 post-infection, shKRT15 #3 failed to do so and 96 h post-infection KRT15 upregulation was similar to that found in the control cells (Supplementary Fig. S3A). VZV replication in shKRT15 #1 and #2 knockdown cells was impaired with no increase in the viral copy number per cell over 96 h (Fig. 3F). This indicated that KRT15 was not only proviral for VZV growth in keratinocytes but also was important and required for VZV growth.

To investigate the role of KRT15 in VZV replication occurring in a model more representative of differentiated skin, we infected control and KRT15 knockdown N/TERT keratinocytes-derived organotypic rafts. As expected, in uninfected control rafts, KRT15 expression was limited to the basal layer (Fig. 3G, top left panel). In contrast, post-VZV infection KRT15 was detected also in the upper layer, with highest expression at the areas of VZV replication, indicated by gE positive expression (Fig. 3G right three upper panels). Importantly, similar to our results with cells growing in monolayer, knockdown of KRT15 (bottom left panel) also inhibited VZV replication in organotypic rafts and the detection of gE (Fig. 3G bottom right two panels).

To further investigate VZV replication in keratinocytes, we next used a VZV expressing mRFP-ORF66 fusion protein, which shows the sites of VZV replication [13]. VZV-infected N/TERTs with KRT15 knockdown displayed greatly decreased mRFP expression, indicating decreased VZV replication, compared to VZV-infected cells expressing scrambled shRNA control (Supplementary Fig. S3B). Taken together, these results strongly indicate that KRT15 is required for VZV replication in skin, with KRT15 upregulation by VZV being proviral.

### The mutations in ORF62 in the vaccine strain vOka prevent KRT15 upregulation

**Fig. 4:**
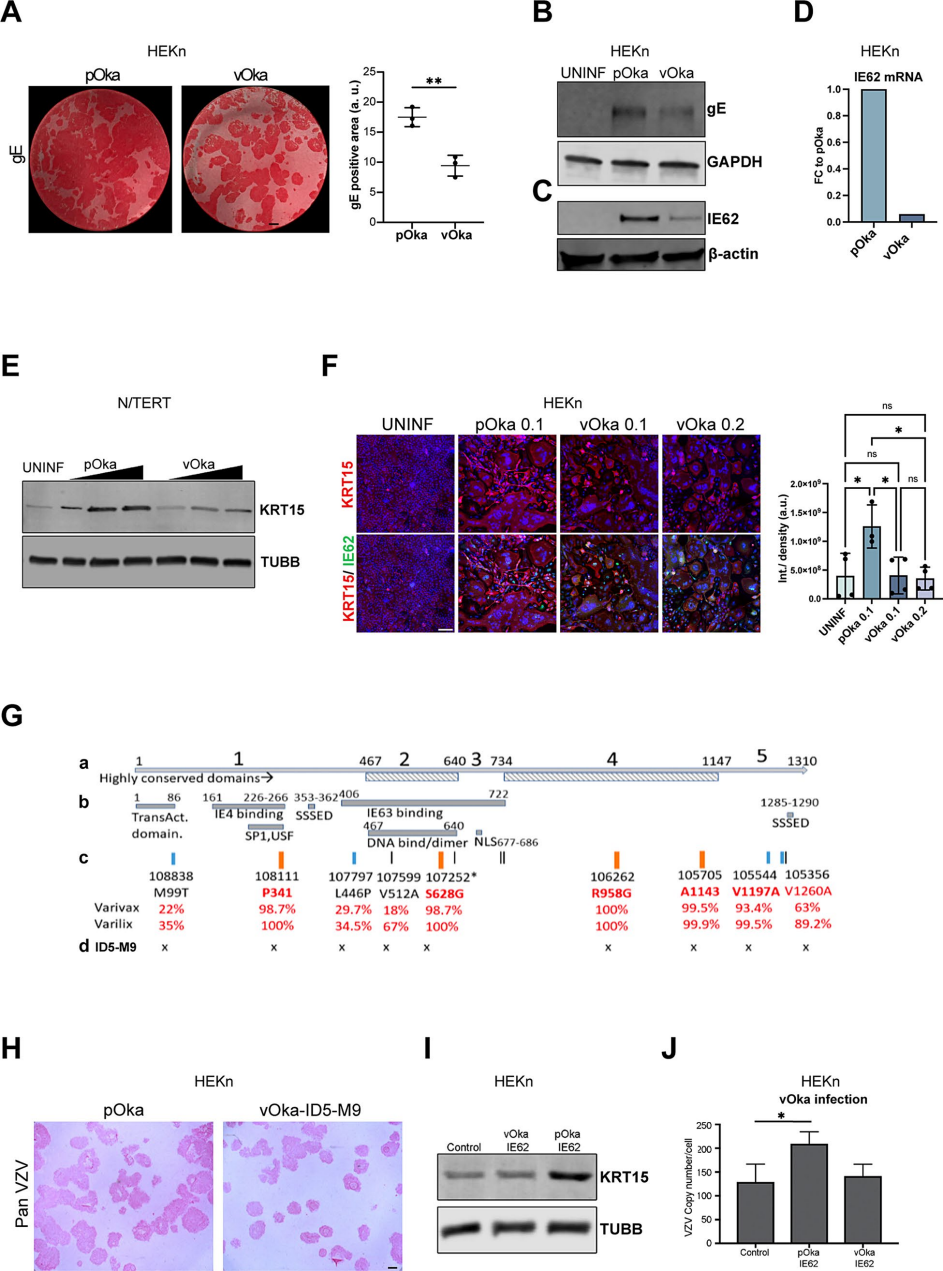
The mutations in ORF62 in the vaccine strain vOka prevent KRT15 upregulation. **(A)** HEKn cells were infected either with pOka or with vOka at MOI 0.1 and analysed 5 days post-infection for gE expression by means of Fast Red staining. Graphs represent average quantification of *n* = 3 biological replicates for surface area gE positive cells ± SD. **(B)** HEKn cells were infected either with pOka or with vOka and analysed 5 days post-infection for gE protein expression by Western Blotting. Data representative of *n* = 2 independent experiments. **(C)** HEKn cells were infected either with pOka or with vOka and analysed 5 days post-infection for IE62 protein expression by Western Blotting. Data representative of *n* = 2 independent experiments. **(D)** HEKn cells were infected either with pOka or with vOka and analysed 5 days post-infection for IE62 mRNA expression by qRT-PCR. Data representative of *n* = 2 independent experiments. **(E)** KRT15 expression levels analysed by Western blotting in N/TERT keratinocytes after infection with increasing (as indicated by a triangle) MOI of the parental (pOka) and vaccine (vOka) VZV strains, with the highest MOI being 0.2. Data representative of *n* = 2 independent experiments. **(F)** HEKn cells were infected either with pOka at MOI 0.1 or with vOka at MOI 0.1 or 0.2 and analysed 5 days post-infection for KRT15 expression. VZV infection is indicated by staining for IE62 protein. Graph shows average quantification of integrated density of KRT15 signal in 4 fields of view for each of the UNINF and vOka conditions and 3 fields of view for the pOka condition. Error bars: SD. Data representative of *n* = 2 independent experiments. **(G) (a)** Schematic of the 5 regions of IE62. High homology regions 2 & 4 are highlighted. **(b)** Known functional/ protein interaction domains mapped to IE62 and their aminoacid co-ordinates. SSED = high serine-acidic tract. NLS = nuclear import signal. **(c)** Mapped vaccine SNPs (with reference to VZV Dumas) are indicated by short vertical lines. Those in orange are near-fixed, and other SNP coding changes (in blue) are indicated. The percentage of vaccine allele SNP in Varivax (Merck) and Varilix (GSK) are indicated. **(d)** The SNP mutations present in vOka-ID5-M9 are indicated. **(H)** HEKn cells were infected either with pOka or with vOka-ID5-M9 and analysed 5 days post-infection for pan VZV expression by means of Fast Red staining. Data representative of *n* = 3 biological replicates. **(I)** KRT15 protein levels from HEKn cells transfected with WT and mutant ORF62 were analysed by Western blotting. Data representative of *n* = 2 independent experiments. **(J)** HEKn cells expressing the pOka or vOka version of the viral ORF62 were infected by the vOka strain and analysed for VZV copy number per cell by qPCR (*n* = 3 independent experiments). Statistical significance was calculated by two-tailed t test **(A)** and one-way ANOVA **(F** and **J)** and denoted by *P < 0.05, **P < 0.01. Scale bar, 100 μm. ns, not significant; SD, standard deviation

The live attenuated vOka vaccine, developed in 1974, has proven safe and effective in the prevention of varicella [14]. The vOka strain is well established to be attenuated for replication in the skin but less so in other target tissues, such as trigeminal ganglia and T lymphocytes [4, 15]. We first confirmed that vOka strain (Varivax, Merck) is attenuated for replication in our model of HEKn infection. We infected HEKn with either pOka or vOka strains at equal MOI (MOI 0.1) and verified equal level of infection by analysis of IE62 and gE proteins expression one day post-infection, when the virus has not yet started replication in HEKn (Supplementary Fig. S4). At five days post-infection the replication of vOka was significantly less compared to pOka, as indicated by the presence of smaller sized plaques, evaluated by the surface area occupied by gE positive cells (Fig. 4A). Consistent with this, the expression of VZV gE and IE62 proteins is lower in vOka-infected compared to pOka-infected HEKn (Fig. 4B and C). This was confirmed at mRNA level for IE62, where gene expression in vOka-infected HEKn is approximately 90% less than in pOka-infected cells (Fig. 4D).

To evaluate how vOka affects the upregulation of KRT15, we infected N/TERT keratinocytes with the pOka or vOka strains at equal levels of virus and monitored KRT15 levels post-infection. We found that, compared to infection with the parental pOka strain, the vOka strain has only a modest effect on KRT15 levels even when cells were infected with the highest MOI possible (MOI = 0.2) (Fig. 4E). This was supported by immunofluorescence analysis of KRT15 expression in HEKn infected with either pOka at MOI 0.1 or with vOka at MOI 0.1 or 0.2. As expected, KRT15 expression was upregulated in the pOka-infected cells, whereas there was no significant change in KRT15 levels between the vOka infections and uninfected cells, even when vOka was used at double the MOI than pOka ) (Fig. 4F). Conversely, overexpression of KRT15 rescued vOka replication, as measured by DNA copy number, to levels similar to those of pOka (Supplementary Fig. S5).

The vOka vaccine is known to be comprised of many genotypes, which carry variable numbers of vaccine single-nucleotide polymorphisms (SNPs) at different frequencies and combinations of alleles. Deep sequencing has shown that 137 SNPs are shared by all vOka vaccines while many additional SNPs are specific to different vaccine preparations [16]. However, only 5 SNPs are present at > 98% frequency in all the vaccines [16] and 4 are located in ORF62 [16–18] (Fig. 4G). Of these 4 mutations, 2 are non-synonymous (NS) resulting in S628G and R958G changes in the ORF62 protein, and these lie in the highly conserved regions 2 and 4, that show near identical protein coding between the equivalent proteins of HSV, VZV, pseudorabies virus and other alphaherpesviruses. The two synonymous mutations are at positions 108,111 (P341) and 105,705 (A1143) [16, 19]. The 4 mutations also occur in almost all vOka vaccine strains recovered from rare cases of post-immunisation rash [17, 20]. Several other vaccine-associated SNPs, both synonymous and non-synonymous, are also found at more variable frequency in ORF62 [21]. Even in vaccine rashes, the vOka vaccine strain remains largely attenuated for replication in skin with fewer lesions, lower viral titers and impaired transmission compared to wild type virus [22]. For this reason, the near-fixed SNPs are thought to play key roles in the attenuation of VZV virulence in the live vaccine. Since vOka is genotypically mixed, we obtained a single variant of vOka, ID5-M9, by plaque purifying the live Varivax vaccine. Sequencing of IE62 from ID5-M9 confirmed that the virus carried all four fixed mutations plus others that have been described for vOka [21] (Fig. 4G). We confirmed that the plaque purified ID5-M9 virus, carrying this IE62, is viable in HEKn culture monolayers but, in keeping with its vOka origin, appears attenuated for replication compared to wild type virus, here demonstrated by smaller sized plaques in primary HEKn (Fig. 4H). We next tested the potential impact of vaccine mutations in IE62 alone by transducing HEKn with a plasmid expressing either pOka or vOka IE62. While expression of pOka ORF62 upregulated KRT15 as expected, expression of the vOka-ID5-M9 ORF62 did not change KRT15 levels (Fig. 4I). Finally, in HEKn infected with vOka (Varivax), exogenous expression of pOka ORF62, but not vOka-ID5-M9 ORF62, rescued vOka replication (Fig. 4J). Taken together, these results show that, unlike wild type pOka IE62, vOka-ID5-M9 ORF62 containing vaccine mutations fails to rescue vOka replication, an effect that mirrors its failure to upregulate KRT15. Given that KRT15 is important for VZV replication in keratinocytes, the possibility seems likely that the failure of vOka to upregulate KRT15 may be a contributing factor to its attenuated replication in skin and thus its success as a vaccine.

## Discussion

We have shown that VZV infection of the skin upregulates keratin KRT15, a type I keratin protein that is normally expressed by epidermal stem cells located in the hair follicle bulge (the earliest site at which VZV can be localised in skin infections [8]) and interfollicular regions of the basal epidermal layer [23]. Because of its expression in hair follicle stem cells, KRT15 has been mooted to be involved in wound healing [24]. Our data strongly implicate a role for KRT15 in VZV replication in the epidermis; shRNA knockdown of KRT15 abrogates VZV replication and thus viral propagation in keratinocytes. Since KRT15 expression is largely confined to epidermal tissues [7, 25], we speculate that upregulation of KRT15 may play a part in VZV tropism for skin.

Our data suggest that VZV upregulation of KRT15 is mediated through the major immediate early transactivating protein IE62 as evident following transduction of IE62 into keratinocytes but not of other proteins that are expressed during the immediate early phase of replication. VZV IE62 is the only regulatory protein that is mutated in the live attenuated VZV vOka vaccine strain used worldwide to prevent varicella in children and shingles in adults [14, 26]. Moreover, four out of the five near-fixation vaccine SNPs, which are found not only in all vaccine batches but also in vOka viruses isolated from vaccine-associated rashes, are located in ORF62 [16, 17, 20]. We show here that reduced vOka replication in keratinocytes is rescued by exogenous wild type virus IE62 expression. This result suggests that vOka-associated IE62 mutations have a role in vaccine attenuation and that a likely mechanism is by preventing the IE62-mediated upregulation of KRT15. However, while our data support the potential importance of IE62 vaccine mutations and their interaction with KRT15 for vOka vaccine attenuation, elucidating precisely the mechanism for this remains to be determined. Furthermore, our findings do not exclude the possible contribution of other vOka mutations to vaccine attenuation. In particular, mutations in ORF0 and ORF31 (glycoprotein B) have also been proposed to reduce replication of vOka and other highly passaged strains in the SCID-hu epithelial mouse model [27–29].

Clearly our evidence indicates KRT15 as important for VZV replication in this specific cell type. We think it highly likely that mutations in vOka vaccine IE62 abrogate its interaction with KRT15 or influence the expression of KRT15, to reduce KRT15-mediated replication in the epidermal tissue, the hallmark of vOka vaccine attenuation. KRT15 may be involved in wound healing and thus upregulated in response to skin injury resulting from VZV infection. As a corollary, we have found in preliminary experiments that exposure of keratinocytes to ionizing radiation (simulating a wounding event) also upregulates KRT15 and that ionizing radiation, similarly to exogenous overexpression of KRT15, increases replication of vaccine vOka in skin but not that of the parental pOka virus (Supplementary Fig. S5). Thus, it may be that the upregulation of KRT15 by injury driven mechanisms is sufficient to overcome the lack of KRT15 induction by vaccine vOka IE62. Further investigation of the possible mechanisms of this operation are underway and are needed to elucidate the pathways more precisely.

## Conclusions

This study provides evidence for a critical importance of keratin 15 in VZV skin replication and, through the interaction with IE62, points to a potential mechanism that may underlie attenuation of the vOka vaccine strain.

## Materials and methods

### Ethics statement and tissue samples

The uninfected skin tissues and HZ skin biopsies analysed were from archival formalin-fixed paraffin-embedded (FFPE) biopsies, obtained with written informed consent under the approval of the East Central London Research Ethics Committee, (10/H0121/39). Immunofluorescence staining shown is representative of *n* = 4 uninfected skin tissues and *n* = 4 HZ skin biopsies (from 4 different patients) examined.

### Cell culture

Primary neonatal human epidermal keratinocytes (HEKn, Life Technologies) and the human keratinocyte telomerase reverse-transcriptase–immortalized (h/TERT-immortalized) N/TERT keratinocytes were cultured on dishes coated with type 1 rat tail collagen solution (Sigma-Aldrich, C3867), in keratinocyte defined media containing epithelial growth factor (EpiLife, Life Technologies) and 1% antibiotics/antimycotics (Thermo Fisher Scientific). The N/TERT keratinocytes used include the cell lines N/TERT-1 and N/TERT-2G, which were derived from clinically normal foreskin tissue [30] and supplied by Dr Rheinwald (Department of Dermatology, Harvard University Medical School, Boston, Massachusetts, USA).

MeWo cells were cultured in MEM (Sigma-Aldrich) containing 10% FBS and 1% antibiotics/antimycotics (Thermo Fisher Scientific).

All the uninfected cells were cultured at 37 °C and 5% CO_2_.

All cells were regularly tested for mycoplasma with mycoplasma PCR detection kit (Sigma-Aldrich).

For skin organotypic rafts generation, N/TERT keratinocytes were seeded on de-epidermalised dermis and successively lifted at the air-liquid interface as previously described [31].

### VZV infections

VZV strains used were pOka, vOka (Varivax, Merck) and the VZV ORF66 RFP strain [32]. ID5-M9 was obtained by plaque purification cloning of virus from the live Varivax vaccine.

To generate cell-free VZV, infected MeWo cells were washed once with ice-cold PBS, collected into PGCS buffer (PBS containing 5% sucrose (w/v), 0.1% monosodium-glutamate (w/v) and 10% FBS) and sonicated (3 times for 15 s with a 15 s interval on ice). Cellular debris was removed by centrifugation (15 min, 1000 g, 4^o^C) and viruses were concentrated using Lenti-X concentrator, which was added to supernatant and incubated for 2 h at 4^o^C before centrifugation (45 min, 1500 g, 4^o^C) to yield viral titres > 1 × 10^5^ pfu/ml.

Alternatively, for some of the experiments of HEKn infection, mitomycin C-treated VZV viruses were used to infect the cells.

Cells were seeded at the number of 1 × 10^5^ cells per well of a 6 well dish and incubated overnight before being incubated with cell-free or mitomycin C-treated virus at the MOI of 0.2 or 0.1. The cells were cultured at 37^o^C for 1 h and then transferred to 34^o^C. HEKn and N/TERTs were treated with calcium chloride (Merck) at the concentration of 1.2 mM to induce keratinocytes differentiation 3 days (HEKn) and 1 or 2 days (N/TERTs) post-infection. The cells were left in culture for 2 further days (5 days total of infection for HEKn and 3 or 4 days total of infection for N/TERTs), before being collected for downstream experimental analyses.

For VZV infection of skin organotypics rafts, the virus was injected intradermally 9 days after air-liquid interface lifting, as previously described in [9].

### Lentiviral transduction

For KRT15 knockdown, keratinocytes were transduced with pGIPZ lentiviruses expressing shRNAs against KRT15 (purchased from UCL Cancer Institute CAGE Facility, clone ids shRNA#1:V3LHS_339599, shRNA#2: V3LHS_339602, shRNA#3:V3LHS_339603).

For KRT15 overexpression, pLenti6.2/V5-DEST™ Gateway™ Vector was used and keratinocytes were then transduced in the presence of 5 mg/mL polybrene.

Plasmids and lentiviruses expressing pOKA or vOKA IE62 were generated by PCR amplification of the whole gene from the respective viruses and cloned into the plasmid vector pGK2 as detailed previously [33] and then subcloned into the lentivirus vector to be under control of the hCMV promoter. Similar approaches were used to express ORF61 and ORF63 under the CMV promoter in pGK2 [34]. In the case lentiviral transduction of N/TERTs with these plasmids, same timing as for VZV infection was followed; the cells were transduced for 3 days before being lysed for subsequent analyses.

In the case of KRT15 knockdown in skin organotypics rafts, siRNA- based gene silencing was used (SMARTpool against KRT15, ON-TARGET plus).

### Drug treatment

The PAA drug was added to the media of cells at the concentration of 5 µg/ml.

### Ionizing radiation

Keratinocytes were subject to1 Gy ionizing radiation 30 min before infection with either pOka or vOka.

### Western blotting

Cells were lysed in either RIPA buffer (Sigma-Aldrich) or 5% SDS lysis buffer supplemented with protease and phosphatase inhibitors. Protein concentration was analysed with Qubit Protein Assays (Thermo Fisher Scientific). Lysates were mixed with 2x Laemmli Sample Buffer (Bio-Rad) containing DTT (0.083 M) and heated to 95 °C for 5 min, before running on a 4–20% Tris-Glycine gel (Bio-Rad) and transferring to a PVDF membrane (Bio-Rad). Membranes were incubated with the indicated antibodies and visualized on a LI-COR Odyssey CLx. For Fig. 1C, the densitometry analysis was performed with the Image Studio™ Software by LICORbio. Densitometry of both KRT15 and β-actin was calculated. The expression of KRT15 normalised to β-actin in each condition is reported as fold change expression of KRT15 in the pOka condition over the expression in the uninfected condition.

Primary antibodies used: KRT15 (1:2,000; Abcam, ab52816), β-actin (1:2,000; Sigma-Aldrich, A1978), GAPDH (1:1,000; Millipore, MAB374), VZV gE (1:500; Santa Cruz, sc-56995), VZV ORF62 (1:1,000; Santa Cruz, sc-17525 or abcam, ab212015), KRT10 (1:500; Biolgend, 905404), TUBB (1:1,000; Merck, T8328). Secondary antibodies used: goat anti-mouse immunoglobulins/HRP (1:3,000; DAKO, P0447), goat anti-rabbit immunoglobulins/HRP (1:3,000; DAKO, P0448), rabbit anti-goat immunoglobulins/HRP (1:3,000; DAKO, P0449); or goat anti-mouse (1:15,000; IRDye^®^ 680RD, LICORbio), goat anti-rabbit (1:15,000; IRDye^®^ 800CW, LICORbio).

### Immunofluorescence

N/TERTs or HEKn grown on type 1 rat tail collagen- coated (Sigma-Aldrich, C3867) coverslips were fixed and permeabilized by 5 min treatment with a solution of 4% paraformaldehyde (Thermo Fisher Scientific) and 0.2% Triton X-100 in PBS.

Cells were blocked with 3% BSA or with a solution of 0.4% fish skin gelatin (Sigma-Aldrich)

and 0.2% Triton X-100 (Sigma-Aldrich) in PBS and incubated with the indicated primary antibody overnight at 4 °C or for 1 h at room temperature, followed by the Alexa Fluor secondary antibody (Life Technologies) at the concentration of 1:500 for 1 h. For staining of skin tissue sections (either skin biopsies or skin organotypics), sections were first deparaffinized in xylene, followed by antigen retrieval in 0.01 M Na Citrate (pH 6). Blocking was performed by incubation with 5% goat serum, before incubation with primary antibodies.

Primary antibodies used: KRT15 (1:500; Abcam, ab52816), ORF9 (gift of William Ruyechan), VZV gE (1:50; Santa Cruz, sc-56995), VZV ORF62 (1:50; abcam, ab212015). For isotype control, the Rabbit IgG (1:500; Cell Signaling, 2729) was used.

Secondary antibodies used: goat anti-rabbit, Alexa Flour 488 conjugate (Life Technologies, A-11008), goat anti-mouse, Alexa Flour 488 conjugate (Life Technologies, A-11001), goat anti-mouse, Alexa Flour 594 conjugate (Life Technologies, A-11005), goat anti-rabbit, Alexa Flour 594 conjugate (Life Technologies, A-11012).

Cells and tissues were mounted in Prolong Gold Antifade Reagent with DAPI (Life Technologies) and visualized on a Zeiss Airyscan 881 or Zeiss LSM710. Images were analysed using the Fiji-ImageJ package [35]. Statistical analyses were performed using Prism (GraphPad Software).

### Immunohistochemistry

The tissue sections were deparaffinized in xylene, quenched with 3% hydrogen peroxide to block endogenous peroxidases, followed by antigen retrieval in 0.01 M Na Citrate (pH 6). Blocking was performed by incubation with 2.5% horse serum and probed with primary antibodies either overnight at 4 °C, or for 1 h at room temperature. The primary antibodies used were: VZV gE (1:50; Santa Cruz, sc-56995) and KRT15 (1:500; Abcam, ab52816). Peroxidase conjugated secondary antibodies (Universal Immpress kit, or Vectastain Universal Elite ABC kit, Vector Laboratories) were used, and DAB substrate kit was used for detection (Vector Laboratories).

### Plaque assays (fast red staining)

Fast Red staining for plaque assays was performed as previously described in [9] and imaged with the ViruSpot Reader (AID GmbH).

### qPCR and qRT–PCR

Genomic DNA for qPCR was extracted using the QIAamp DNA mini-kit (Qiagen).

Cell total RNA was extracted using the RNeasy kit (Qiagen). Approximately 100 to 1000 ng of total RNA was used for cDNA synthesis using the Super-Script III Reverse Transcriptase (Thermo Fisher Scientific). DNA levels were quantified by qPCR using optimised primers and probes, and GoTaq Probe qPCR Master Mix (Promega). mRNA levels were quantified by qRT-PCR using optimized primers and SYBR Green PCR master mix (Applied Biosystems) or TaqMan technology (Thermo Fisher Scientific).

## Electronic supplementary material

Below is the link to the electronic supplementary material.

Supplementary Material 1

## Data Availability

No datasets were generated or analysed during the current study.

## Abbreviations

VZV: Varicella-zoster virus
KRT15: Keratin 15
PAA: Phosphonoacetic acid
IE: Immediate early
HEKn: Neonatal human epidermal keratinocytes
SNPs: single-nucleotide polymorphisms
NS: Non-synonymous
