# Supplementary material for: Upregulation of keratin 15 is required for varicella-zoster virus replication in keratinocytes and is attenuated in the live attenuated vOka vaccine strain
Source: Virol J. 2024 Oct 9;21:253. doi: 10.1186/s12985-024-02514-8 (PMC11465976; doi:10.1186/s12985-024-02514-8)

Supplementary material:

**Supplementary Fig. S1:**

KRT15 expression as visualised by immunofluorescence in N/TERT keratinocytes infected with pOka, which was detected with antibodies to the late-regulated ORF9 protein. Images representative of n=2 independent experiments.

**Supplementary Fig. S2:**

N/TERT keratinocytes were infected in the presence or absence of PAA, inhibitor of the viral DNA polymerase. 96 hours post-infection cells were analysed for the expression levels of the indicated proteins by Western blot.

**Supplementary Fig. S3:**

Expression levels of KRT15 post VZV infection in KRT15 knockdown N/TERTs. **(A)** KRT15 knockdown cells were infected by VZV and harvested at the indicated time points and analysed for KRT15 expression levels using qRT-PCR. **(B)** GFP-expressing N/TERTs were transduced either with lentivirus carrying shRNA #2 for KRT15 or shRNA control and infected with RFP-tagged VZV virus.

**Supplementary Fig. S4:**

Expression of IE62 and gE proteins as evaluated by integrated density (int./density) analysis of immunofluorescence staining for IE62 and gE after 1 day of pOka or vOka infection of HEKn. Bars: average. Error bars: SD (n= 5 fields of view).

Statistical significance was calculated by two-tailed t test. SD, standard deviation; ns, not significant.

**Supplementary Fig. S5:**

Prior to infection with pOka or vOka, HEKn were exposed to ionizing radiation (IR) or infected with lentiviruses overexpressing KRT15. VZV copy number per cell was measured by qPCR.

Error bars: SD (n=3 independent experiments).

Statistical significance was calculated by one-way ANOVA. SD, standard deviation.


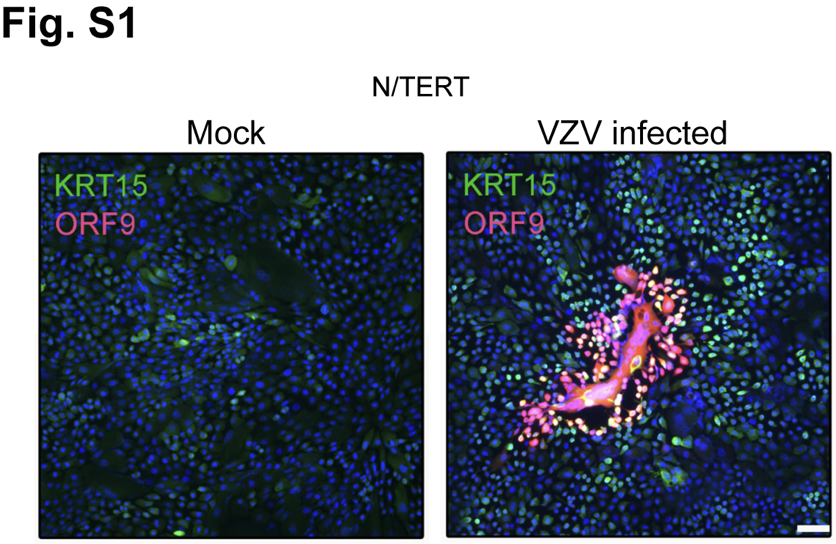


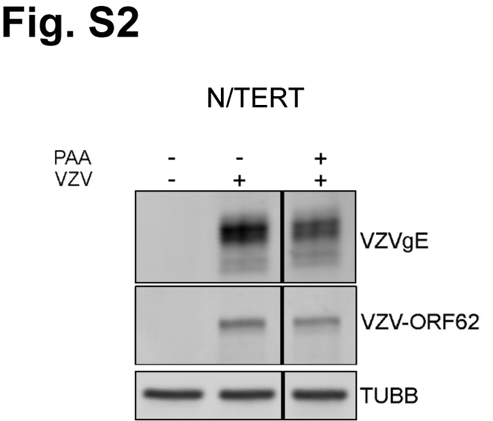


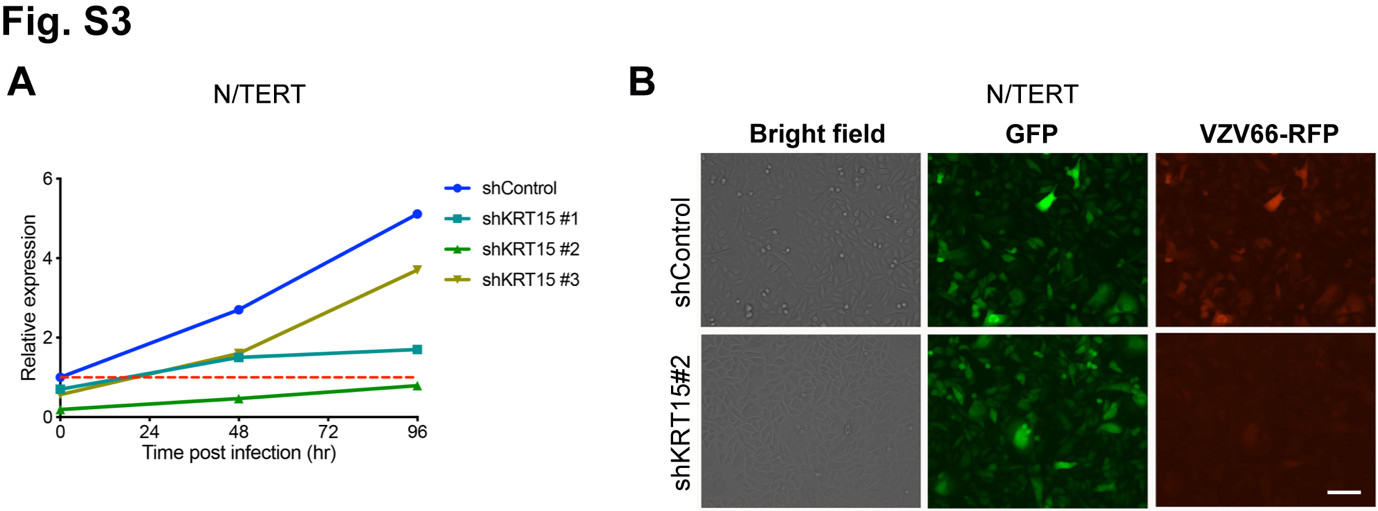


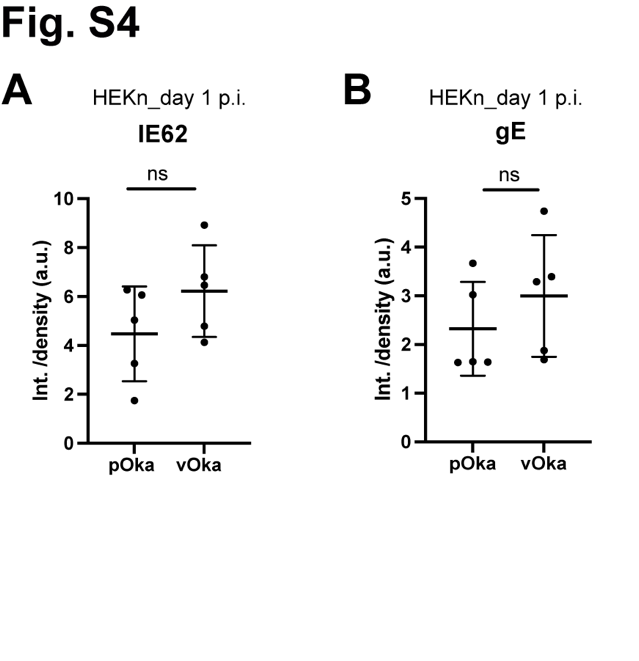


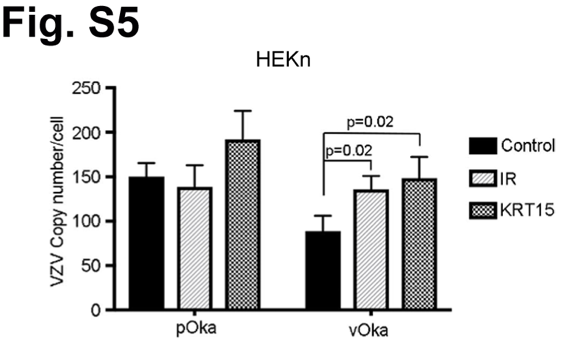

Supplement: Supplementary file 1 — Supplementary Material 1 [file 12985_2024_2514_MOESM1_ESM.docx]
